# Development, testing, and application of a mathematics learning scale of self-direction

**DOI:** 10.3389/fpsyg.2023.1145442

**Published:** 2023-05-12

**Authors:** Chia-Hui Lin, Chang-Hua Chen

**Affiliations:** ^1^Office of Teacher Education and Careers Service, National Taichung University of Education, Taichung, Taiwan; ^2^Graduate Institute of Science Education, National Changhua University of Education, Changhua, Taiwan

**Keywords:** learning scale, mathematics education, self-directed learning (SDL), self-direction, self-reguated learning

## Abstract

Many countries’ curriculum reforms focus on developing the next generations’ competencies of self-directed learning (SDL) to address rapid social changes and sustainable environmental development. Taiwan’s curriculum reform corresponds with the global trend in education. The latest curriculum reform, which proposed a 12-year basic education, was implemented in 2018 and included SDL explicitly in its guidelines. The reformed curriculum guidelines have been followed for over 3  years. Thus, it is necessary to conduct a large-scale survey to examine its impact on Taiwanese students. However, existing research instruments help provide a generalized analysis of SDL and have yet to be designed specifically for SDL of mathematics. Therefore, we developed a mathematics SDL scale (MSDLS) and examined its reliability and validity in this study. Subsequently, MSDLS was utilized to investigate Taiwanese students’ SDL of mathematics. The MSDLS consists of four sub-scales with 50 items. It has acceptable reliability, validity, and measurement invariance across gender and grade groups. The MSDLS was administered online to 5,575 junior high school students, and 5,456 valid responses were collected. The findings highlight the gender and grade differences in SDL of mathematics. Male students are higher than female students in many factors. It is noted that the SDL in mathematics does not increase with grade. In sum, the MSDLS is a helpful instrument for examining secondary school students’ SDL of mathematics.

## 1. Introduction

In response to the rapid changes in society and the environment in the 21st century, countries around the world are pursuing quality education and social justice. They consider what and how the next generation should learn (Senge et al., 2000). For example, the New Zealand Curriculum identifies five key competencies. Among the five key competencies, managing self means that students should establish personal learning goals, set high standards, make plans, manage projects, and have strategies for meeting challenges (New Zealand Ministry of Education, 2007). Hong Kong has been promoting the “Learning to Learn” curriculum reform since 2001, updated the curriculum framework to “Learning to Learn 2.0” in 2017, and added self-directed learning (SDL) abilities (H.K. Curriculum Development Council, 2017), hence enabling students to become independent and self-directed learners. The Organization for Economic Cooperation and Development (OECD) has included self-direction in the mathematical assessment framework of the Program for International Student Assessment (PISA) as a key 21st century skill (OECD, 2018). In other words, SDL has gradually become the common language for curriculum reform in many countries or regions.

Taiwan’s curriculum reform corresponds with the global trend in education. This is the first time that SDL has been included in the Curriculum Guidelines of 12-Year Basic Education: General Guidelines (referred to as general guidelines) and SDL is considered a prerequisite for lifelong learning and whole-person education (National Academy for Educational Research, 2018). The general guidelines use the core competencies as the basis of curriculum development. These competencies are divided into three broad dimensions: spontaneity, communication and interaction, and social participation. SDL functions as the purpose and process of the development of core competencies. As an element of core competencies, SDL demonstrates learners’ knowledge, skills, and attitudes related to learning after completing 12-year basic education and lays the foundation for lifelong learning and career development. In addition, SDL becomes an indispensable process for the development of core competencies. Students set learning goals and adopt strategies to achieve these goals through SDL. In this process, they continuously evaluate the gap between the current scenario and the goals and revise their strategies to reduce the gap and move towards the goals.

The general guidelines encourage elementary and secondary schools to integrate SDL into school-based curricula (National Academy for Educational Research, 2018, pp. 11, 13) and alternative learning periods (pp. 21, 28). Schools and teachers should guide students in learning how to learn, including general learning strategies, domain learning strategies, and metacognitive strategies (National Academy for Educational Research, 2018, p. 48). High schools should include the spirit and practice of SDL in school-based curriculum development and key items of school evaluation and school visits. In Taiwan’s curriculum reform, the general guidelines serve as a guiding document of curriculum development and guide the construction and design of domain-specific curriculum guidelines (referred to as domain guidelines). Therefore, the spirit of SDL is explicitly included or incorporated into the domain guidelines. For example, the core competencies of the mathematics curriculum guidelines at the junior high school level are “students should be able to identify the connection between real-life problems and mathematics, develop problem-solving strategies from multiple perspectives, and apply them in real-life scenarios” (Ministry of Education, 2018, p. 3). Including SDL in the mathematics curriculum guidelines prepares students for university study and career development and addresses the problem of low motivation, low self-confidence, and negative attitudes of Taiwanese students in previous international mathematics learning achievement assessments.

Although Taiwan’s curriculum reform emphasizes SDL, SDL is not clearly defined in the general and domain guidelines. Furthermore, although PISA (OECD, 2018) includes self-direction in the 2022 mathematical literacy assessment framework, it does not provide the definition and related aspects of SDL in mathematics. Therefore, this study aimed to construct a mathematics SDL scale and examine its reliability. This study conducted a survey in Taiwan and examined the differences in SDL in mathematics across demographic variables. As the curriculum reform of 12-year basic education was implemented more than 3 years ago, it is necessary to develop a research instrument to investigate the current scenario of SDL in mathematics among Taiwanese students. This paper aims to clarify the concept of SDL in mathematics in the context of curriculum reform and propose a measurement tool to facilitate the understanding and discussion of this 21^st^ century skill in the academic world. From a practical perspective, this paper identifies possible problems in implementing the new curriculum guideline and proposes some educational policy recommendations.

Thus, the objectives of this investigation are dual-fold: (a) fashioning a mathematical learning scale of self-direction and evaluating its reliability and validity and (b) exploring the SDL variance in mathematics with respect to gender and scholastic levels.

## 2. Literature review

### 2.1. Definitions of self-directed learning (SDL)

SDL has received much academic interest over the past four decades (Panadero, 2017) and has had various definitions (de Bruin and van Merriënboer, 2017; Brandt, 2020). Knowles (1975) defined SDL as a means for individuals to actively diagnose their own learning requirements, set learning goals, find relevant learning resources, select and apply appropriate learning strategies, and evaluate learning outcomes. SDL is often confused with self-regulated learning (SRL), but SDL has been considered a broader concept encompassing SRL (Saks and Leijen, 2014). The general guidelines also consider SRL as a sub-process of SDL and emphasize its development in elementary schools (National Academy for Educational Research, 2018). SRL provides learners with the self-direction to use their mental abilities to regulate their learning behaviors, transform their mental abilities into academic performance, and perform self-reflection after learning tasks, thereby preparing themselves for the next learning task (Zimmerman, 2008). Therefore, SDL is closely related to metacognition. Some scholars, such as Mevarech et al. (2018), believed that the two terms can be considered synonymous in mathematics classrooms.

Although academic perspectives on SDL are divergent, commonalities exist among the views that focus on how learners organize, guide, and monitor individual learning activities. Steffens (2015) believed that the object of SDL theory is not the learning itself, and it is closer to a meta-theory focusing on how to learn. It involves learning motivation, learning strategies, learning regulation, and learning resource management (National Academies of Sciences, Engineering, and Medicine, 2018). Zimmerman's (2000) believed that the key to SDL is self-regulation, which is a three-stage cycle that includes motivation and self-efficacy, strategy development and implementation, and self-monitoring and self-adjusting. Self-directed learners demonstrate excellent self-control abilities in cognitive and behavioral strategies, and these abilities are reflected in their active participation in learning activities and excellent academic achievements (Schunk and Rice, 1987, 1991). In addition, self-directed learners can manage learning resources and seek help when they encounter challenges (Schunk, 2012).

The mathematics education community has been exploring SDL for a long time, focusing on planning, monitoring, evaluating, and reflecting in problem-solving. In the influential book, “How to Solve It,” Polya (1957) proposed a four-step model for problem-solving: understanding the problem, developing a problem-solving strategy, implementing the strategy, and reviewing the solution. First, the problem solver should understand the problem, identify the type of problem by its conditions, devise a problem-solving plan, select a problem-solving strategy, implement the problem-solving plan, and monitor the effectiveness of the strategy. After obtaining the solution, the problem solver should check whether the solution is correct and whether a better solution exists. Schoenfeld's (1985) extended Polya’s view of problem-solving by proposing a six-step model: reading the problem, analyzing the problem, exploring the known conditions and goals of the problem, devising a problem-solving plan, implementing and monitoring the plan, and evaluating the solution. He believed that mathematical problem-solving includes four elements: knowledge resources (resources), strategy exploration (heuristics), control (control), and belief systems (belief systems). The control is self-regulation, which is the key to the mathematical problem-solving process (Schoenfeld, 1992). Schoenfeld's (1985) research became the theoretical basis for many subsequent studies related to SDL in mathematics (Mevarech et al., 2018).

### 2.2. Previous views on SDL

SDL covers a wide range of concepts. Some scholars discuss it from a holistic perspective and others focus on a particular direction (Brandt, 2020). Bandura's (1977) social cognitive theory proposed the concept of SDL at an early stage. He believed that SDL is the result of individuals’ external behaviors and the process by which individuals monitor, evaluate, and modify their cognition, motivation, emotions, and behaviors. Some scholars (such as Winne, 1996) focused on cognitive processes, such as whether learners have the necessary knowledge, abilities, and strategies to achieve goals. Self-directed learners demonstrate excellent self-control abilities in cognitive and behavioral strategies, and these abilities are reflected in their active participation in learning activities and excellent academic achievements (Schunk and Rice, 1987, 1991). Some scholars (such as Corno, 2001) indicated that many students fail to perform SDL not because they lack the relevant knowledge and ability, but because they lack the willingness to use it in the classroom. Therefore, motivation and attitude have an important role in SDL. When learners enter a classroom and begin learning, their personal goals, self-efficacy, values, and emotions determine how they approach the task (namely learning strategies) and how much effort they apply to it (Schunk and Mullen, 2013; Wiliam, 2018). Most studies on SDL use self-report scales (Saks and Leijen, 2014), and the commonly used scales include the self-directed learning readiness scale (SDLRS) developed by Guglielmino (1977) and the motivated strategies for learning questionnaire (MSLQ) developed by Pintrich et al. (1991). The SDLRS and MSLQ assess general SDL skills. They have been translated into multiple languages and implemented in many countries and have excellent reliability and validity.

Many studies indicated SDL abilities can be acquired and improved (Gabrielle et al., 2006; Amey, 2008; Dignath et al., 2008). Chen et al. (2021) conducted a large-scale survey using SDLRS and observed that the 12-year basic education curriculum assisted in improving the SDL readiness of high school students. Teaching students SDL skills can enhance their mathematical reasoning (Schoenfeld, 1992; Mevarech and Kramarski, 2014). Note that although SDL is considered a domain-general skill, it can only be effectively taught and developed in a domain-specific context, e.g., mathematics (Kirschner and Hendrick, 2020; Schunk, 2020). The more mathematical knowledge and strategies one has, the more effective one can self-monitor and self-regulate in mathematics learning (Schoenfeld, 2014). Developing and applying SDL skills in domain-specific learning, such as using general learning strategies and problem-solving strategies in mathematics learning, can improve mathematics learning performance and facilitate learning transfer (Schunk, 2012). Research demonstrates that K-12 students and adults who plan, monitor, evaluate, and reflect when solving mathematical problems perform better in problem-solving (Stillman and Mevarech, 2010). In mathematics learning, self-regulation is highly correlated with academic achievement, particularly in solving complex, non-routine, and unfamiliar mathematical problems (Mevarech et al., 2018). Gender and grade differences have been observed in SDL. Females have a lower self-efficacy in solving mathematical problems than males and often attribute low performance to uncontrollable factors (low ability and difficult tasks) (Vermeer et al., 2000). SDL abilities may increase as grade level increases (Chen et al., 2021), but some dimensions (such as motivation and self-confidence) may decrease as grade level increases (Mok et al., 2007).

In sum, the importance of SDL in mathematics education is evident, as these skills have been identified as crucial factors in improving student performance. It is noted that there need to be more well-developed learning scales to assess and measure self-directed learning skills in mathematics education effectively. While existing self-report scales, such as SDLRS and MSLQ, have been widely used and validated, they primarily assess general SDL skills. This research gap presents an opportunity for researchers to create targeted and reliable instruments that will enhance our understanding of the role of self-directed learning in mathematics and support educational strategies to foster these skills in students.

## 3. Research methods

### 3.1. Study participants

This study was divided into a pretest sample and a final sample. The pretest sample was used for item analysis and exploratory factor analysis (EFA). The final sample was used for confirmatory factor analysis (CFA), cross-validation, and measurement invariance tests. Crocker and Algina (2006) suggested that the number of participants should be at least five times the number of items in item analysis. If the number of participants is 10 times the number of items, the results will be more stable. Therefore, this study invited students from a private junior high school that accepts applications from all students in Taiwan to complete the online survey and used the data as a pretest sample. As a NAER partner school, it has been implementing an SDL curriculum since the development of the general guidelines. Additionally, it is the target of NAER’s survey on the effectiveness of the implementation of the new curriculum guideline and it is representative. Students from this school could answer the online survey from September to mid-October 2021. There were 681 valid responses, and the return rate was 99.5%.

The final sample was obtained from six other NAER partner schools, which are also the targets of NAER’s survey on the effectiveness of the implementation of the new curriculum guideline. These schools have different sizes and are in the northern, central, southern, and eastern regions of Taiwan; hence, they are regionally representative. The researcher invited all students to complete the online survey, but some schools only permitted seventh and eighth graders to participate in the survey because the ninth graders were preparing for the high school entrance exams. A total of 5,575 students completed the online survey between the end of October and December 2021, and 5,456 valid responses were collected. The return rate was 97.8%.

### 3.2. Scale development and validation

**Figure 1:**
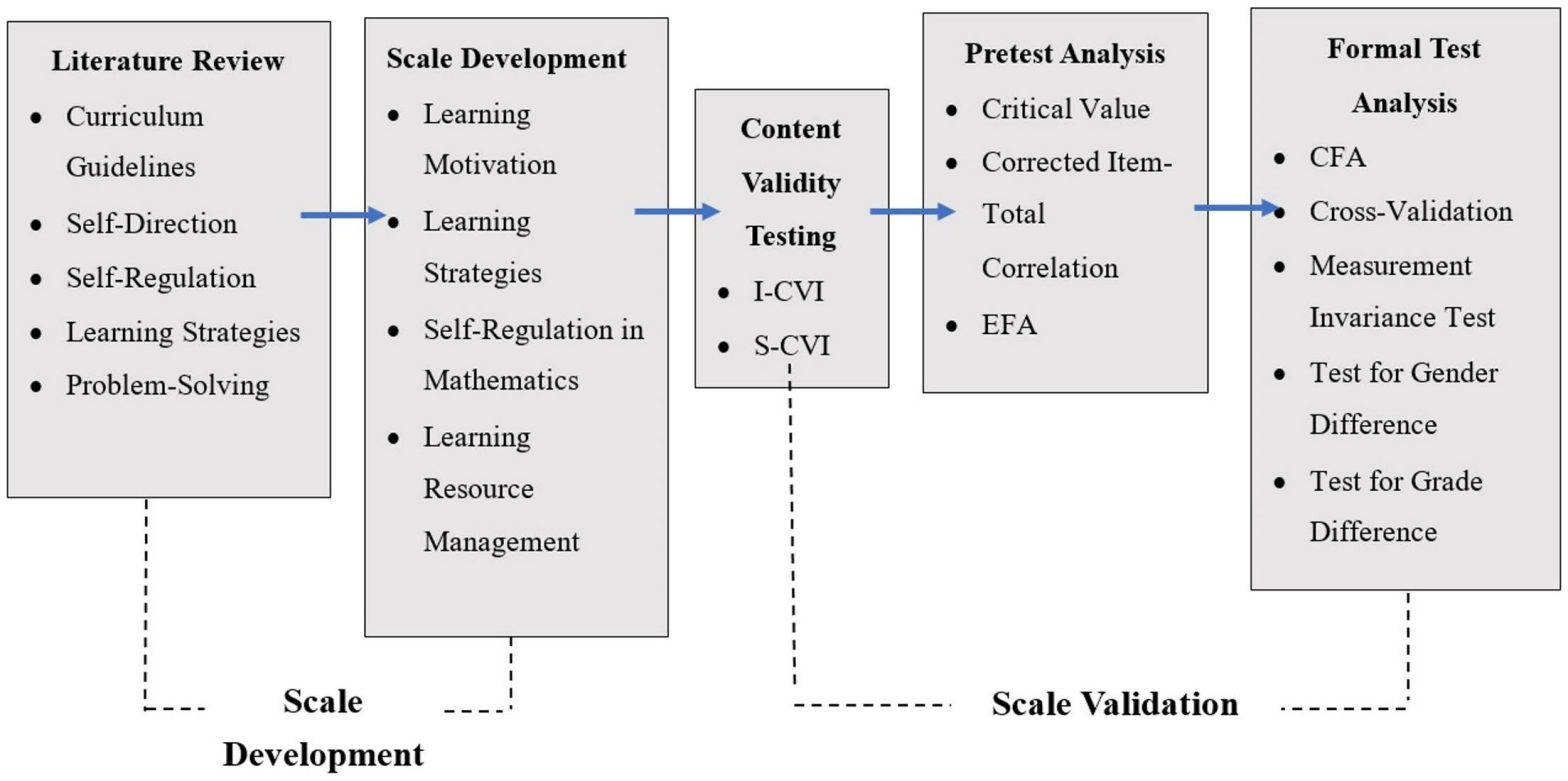
Framework for the development and validation of the mathemetics SDL scale.

The scale was developed in two stages: scale development and scale validation. The first stage involved constructing the items of the scale based on the curriculum guideline document and literature review of SDL. The second stage was divided into three sub-stages: content validity testing, pretest analysis, and formal test analysis, as shown in Figure 1.

#### 3.2.1. Scale development

Because SDL is both domain-specific and cross-disciplinary, this study first defined the SDL in junior high school mathematics based on Schoenfeld's (1985) and Zimmerman's (2000) theories and the 12-year national mathematics curriculum guidelines. Subsequently, this study used the Delphi technique to examine and modify the definition. Delbecq et al. (1975) suggested that the number of experts in the Delphi technique should be 15 to 30 when the homogeneity of experts is high and can be reduced to five to ten when the homogeneity is low. However, if the number of experts in the Delphi technique is more than ten, the error of the expert group is minimized, and the group is most credible (Dalkey, 1969). Therefore, this study invited 15 experts, including the general guideline development committee, the mathematics curriculum guideline development committee, mathematics education scholars, mathematicians, scholars who conduct tests and assessments, and expert mathematics teachers to participate in the expert group of the Delphi technique. These experts provided advice from various professions and perspectives. After two Delphi techniques, all experts agreed that the definition of SDL in mathematics at the junior high school level is, “A junior high school student who has the SDL ability in mathematics is motivated to learn, can assess his/her learning needs, set learning goals, use strategies and resources to help achieve learning goals, identify and adjust his/her learning status, solve mathematical problems effectively, and reflect on the effectiveness of mathematical problem-solving strategies.” Students who have completed three-year junior high school mathematics learning of the 12-year basic education should possess SDL competencies.

After establishing the definition of SDL in mathematics, the researcher used the definition to develop the scale from four dimensions: learning motivation, learning strategies, mathematics self-regulation, and learning resource management. According to experts’ recommendations, the researcher adopted a four-point Likert scale that included strongly agree, agree, disagree, and strongly disagree. The researcher referred to SDL-related literature and scales (such as SDLRS and MSLQ; Mevarech and Kramarski, 2014), selected items from international surveys such as TIMSS and PISA, and modified the items. Additionally, the researcher compiled some items based on the definition to expand the item database of the scale.

#### 3.2.2. Scale validation

In this stage, this study examined the content validity. Referring to Cheng et al. (2010), the researcher invited the 15 experts to evaluate each item individually and independently using a four-point Likert scale. The scoring criteria were as follows: (i) Appropriateness: this item reflects the definition of SDL in mathematics and is appropriate for assessing junior high school students and (ii) Clarity: this item is clear and easy to understand for junior high school students. After evaluating each item, the experts provided a score of 4 for the item being highly appropriate, clear, and precise, 3 for the item being appropriate and requiring minor revisions, 2 for the item being less appropriate and requiring major revisions, and 1 for the item being inappropriate and should be deleted. During the examination and grading process, the experts provided advice for the revision of the items if necessary. After the experts rated the items, the researcher calculated the content validity index (CVI) based on their ratings. The CVI value of each item (I-CVI) is the number of experts scoring three or more divided by the total number of experts. The CVI value of the scale (S-CVI) is the average of the I-CVI values within the scale (Polit and Beck, 2006). After the scale and the items passed the content validity test, a first draft of 68 items was completed.

This study used 68 items in the pretest and conducted item analysis and EFA. The researcher removed items with low-quality scores from the first draft based on the critical values and corrected item-total correlation (Spector, 1992) to ensure the quality of the items on the scale. Subsequently, this study conducted EFA, extracted factors using the principal axis factoring (PAF) method, and determined the number of factors based on the Kaiser eigenvalues greater than one and Cattell’s scree plot. Because the factors were correlated with each other, in terms of the factor rotation methods, this study used the Promax method of oblique rotation to perform the rotation. The selection criterion was that the factor loading of the pattern matrix was greater than 0.40 (Wu, 2012), and 50 items were selected.

Subsequently, the researcher conducted the formal test, CFA, cross-validation, and measurement invariance tests across gender and grade. This study used the structural equation models to construct measurement models for the observed variables based on the EFA results and analyzed the results using AMOS 22.0 software and the maximum likelihood (ML) estimation method to confirm the composite reliability and convergent validity of the data. However, the aforementioned model may be rejected in CFA owing to the large sample size and model complexity (Bentler and Bonett, 1980; Marsh and Hocevar, 1985; Marsh et al., 1988). The scale consisted of 50 items in the formal test, and Bentler and Chou (1987) suggested that when the population distribution is unknown, the number of participants for the CFA should be larger than 10 times the number of items. Therefore, we randomly selected 1,014 students from the 5,456 samples from six schools based on the proportion of students in each school and randomly divided them into two groups. The first group was selected as the calibration sample (*N*_1_ = 507) for CFA. AMOS 22.0 was used to examine the degree of consistency between the factor structure and the theory. For the CFA, all factors were allowed to covary (The Pearson correlation coefficient matrix for all study items is available at https://doi.org/10.5281/zenodo.7094563). After the model was fitted well, the second group was used as the validation sample (*N*_2_ = 507) for cross-validation. To examine gender and grade differences, this study tested the measurement invariance of the scale across gender and grade using cohort analysis.

### 3.3. Analysis of gender and grade differences

This study examined gender and grade differences after measurement invariance was established. This study used the independent sample *t*-test to examine the latent mean difference across gender and used the analysis of variance to examine the latent mean difference across grades. If significant differences were observed between students in different grades, further comparisons were made. The data were analyzed using the software SPSS 22.0.

## 4. Research findings

### 4.1. Content validity test

This scale consisted of four subscales: learning motivation, learning strategies, mathematical self-regulation, and learning resource management. After the first round of expert evaluation, the I-CVI values of the items on the scale were between 0.6 and 1, and the S-CVI was 0.9. Polit and Beck (2006) suggested that an I-CVI greater than 0.78 and an S-CVI greater than 0.9 are acceptable when the number of experts is large. Although the experts approved the content validity of the first draft of the scale, some items required to be deleted or revised. The researcher deleted the items with an I-CVI below 0.78 and revised some items according to experts’ suggestions. After the second round of expert review, the study calculated the CVI values. The S-CVI was 0.97, the CVI values for each subscale were between 0.96 and 0.99, and the CVI values for each item were between 0.87 and 1.00. The items that passed the content validity test were included in the first draft of the scale to conduct the pretest.

### 4.2. Item analysis

Item analysis refers to the process of selecting appropriate items based on critical values and corrected item-total correlation (DeVellis, 1998). The critical value method was used to divide the pretest sample into high-score and low-score groups (27% each). Subsequently, the two groups were used as independent variables, and the scores of individual items were used as dependent variables for an independent sample t-test. The scores of discriminating items should be significantly different in the two groups. In this study, the significance level was set at *α* = 0.05. The results showed that the scores of all items were statistically significant (*p* < 0.05), meaning that all items on the scale demonstrated good discriminatory power. The corrected item-total correlation was calculated using the Pearson product–moment correlation coefficient between each item and the total score of the subscale (excluding the score of the item). The criterion for selecting items for this study was that the corrected item-total correlation coefficient must be above 0.3. The results showed that the correlation coefficients of all items were above 0.3, which meant that they were homogeneous. In other words, all items satisfied the criterion in terms of discriminatory power and corrected item-total correlation coefficient. Therefore, all items were retained for the EFA.

### 4.3. Exploratory factor analysis (EFA)

To confirm whether the data were suitable for factor analysis, this study used the Kaiser–Meyer–Olkin measure of sampling adequacy (KMO) to examine the correlation coefficients between the variables and used Bartlett’s spherical test values to examine whether the correlation coefficients in the correlation coefficient matrix were significantly higher than zero. The results showed that the KMO values of each subscale of the junior high school mathematics SDL scale were above 0.60. The Bartlett’s spherical test values were 15433.47, 5593.17, 19157.58, 606.57, and 5379.56, which were all significant (*p* < 0.001) (Table 1). This indicated that the sample and subscales were suitable for the factor analysis.

**Table 1:** KMO and Bartlett’s spherical test values for each subscale of the mathematics SDL scale.

		Learning motivation	Cognitive strategies	Mathematical self-regulation	Learning resource management
KMO measure of sampling adequacy		0.959	0.915	0.977	0.927
Bartlett’s spherical test	*χ* ^2^	15433.47^***^	50.38.42^***^	15554.24^***^	5379.56^***^
	*df*	276	36	210	45
	*p*	<0.001	<0.001	<0.001	<0.001

^***^p < 0.001.

Chen and Chang (2007) indicated that because the factors are correlated with each other, it is not possible to list the commonality of the items and the percentage explained by each factor. Therefore, only the factor loadings are presented in this paper.

#### 4.3.1. Learning motivation pretest subscale

As shown in Table 2, the learning motivation pretest subscale included five factors. For Factor 1, four items satisfied the standard with factor loadings between 0.85 and 0.99, and the factor was called self-efficacy based on the theme of the items. For Factor 2, three items satisfied the standard with factor loadings between 0.50 and 0.64, and the factor was called the identified motivation based on the theme of items. For Factor 3, four items satisfied the standard with factor loadings between 0.57 and 0.92, and the factor was called extrinsic motivation (work values) based on the theme of items. For Factor 4, four items satisfied the standard with factor loadings between 0.47 and 0.83 and the factor is named intrinsic motivation based on the theme of items. For Factor 5, three items satisfied the standard with factor loadings between 0.48 and 0.80, and the factor was called extrinsic motivation (achievement-oriented) based on the theme of items.

**Table 2:** Summary of factor analysis for the learning motivation pretest Subscale.

Item no.	Factor 1	Factor 2	Factor 3	Factor 4	Factor 5
Q22	0.99	−0.05	0.01	−0.09	0.05
Q24	0.90	0.03	0.07	−0.06	−0.11
Q23	0.86	−0.04	−0.04	0.09	−0.02
Q21	0.85	−0.04	−0.02	0.06	0.07
Q17	0.05	0.64	0.01	−0.03	0.10
Q18	0.09	0.54	0.11	0.05	0.07
Q14	0.12	0.50	0.15	−0.09	0.20
Q7	−0.07	0.03	0.92	−0.01	−0.04
Q8	−0.02	−0.05	0.92	−0.08	0.09
Q9	0.05	−0.05	0.75	0.14	0.05
Q10	0.11	0.17	0.57	0.11	−0.17
Q2	0.08	0.04	−0.01	0.83	−0.09
Q1	0.24	−0.08	−0.03	0.79	0.01
Q4	0.27	−0.07	−0.06	0.73	0.05
Q6	0.04	0.10	0.14	0.47	0.04
Q13	−0.04	0.07	−0.06	−0.03	0.80
Q12	0.05	0.06	0.04	0.02	0.65
Q11	0.03	−0.04	0.35	−0.02	0.48
Factor name	Self-efficacy	Identified motivation	Extrinsic motivation (Work values)	Intrinsic motivation	Extrinsic motivation (Achievement-oriented)

#### 4.3.2. Learning strategy pretest subscale

As shown in Table 3, the learning strategy pretest subscale consisted of three factors. For Factor 1, four items satisfied the standard with factor loadings between 0.53 and 0.73, and the factor was called refined strategies based on the theme of items. For Factor 2, three items satisfied the standard with factor loadings between 0.56 and 0.95, and the factor was called rehearsal strategies based on the theme of items. For Factor 3, two items satisfied the standard with factor loadings between 0.61 and 0.87, and the factor was called critical thinking strategies based on the theme of items.

**Table 3:** Summary of factor analysis for the learning strategy pretest subscale.

Item no.	Factor 1	Factor 2	Factor 3
Q28	0.73	0.07	0.04
Q29	0.68	0.24	−0.03
Q31	0.53	0.04	0.22
Q25	−0.03	0.95	−0.04
Q26	0.33	0.59	−0.05
Q27	−0.10	0.56	0.22
Q32	0.00	0.06	0.87
Q33	0.29	−0.01	0.61
Factor name	Refined strategies	Rehearsal strategies	Critical thinking strategies

#### 4.3.3. Mathematics self-regulation strategy pretest subscale

As shown in Table 4, the mathematics self-regulation strategy pretest subscale consisted of two factors. For Factor 1, ten items satisfied the standard with factor loadings between 0.44 and 0.98, and the factor was called self-regulation in problem-solving based on the theme of the items. For Factor 2, seven items satisfied the standard with factor loadings between 0.54 and 0.77, and the factor was called SRL in mathematics based on the theme of items.

**Table 4:** Summary of factor analysis for the mathematics self-regulation strategy pretest subscale.

Item no.	Factor 1	Factor 2
Q49	0.98	−0.17
Q50	0.87	−0.01
Q47	0.85	−0.02
Q48	0.80	0.03
Q51	0.79	0.03
Q54	0.74	0.06
Q53	0.74	0.11
Q45	0.74	0.11
Q52	0.55	0.27
Q46	0.44	0.37
Q35	0.00	0.77
Q38	0.05	0.71
Q40	0.16	0.70
Q42	0.19	0.69
Q41	0.18	0.67
Q43	0.32	0.54
Q37	0.25	0.54
Factor name	Self-regulation in problem-solving	SRL in mathematics

#### 4.3.4. Learning resource management pretest subscale

As shown in Table 5, the learning resource management pretest subscale consisted of two factors. For Factor 1, four items satisfied the standard with factor loadings between 0.48 and 0.95, and the factor was called time management and study environment based on the theme of items. For Factor 2, three items satisfied the standard with factor loadings between 0.47 and 0.62, and the factor was called interpersonal interaction and help-seeking based on the theme of items.

**Table 5:** Summary of factor analysis for the learning resource management pretest subscale.

Item	Factor 1	Factor 2
Q57	0.95	−0.17
Q56	0.83	0.05
Q55	0.79	0.03
Q59	0.48	0.23
Q63	0.16	0.62
Q62	0.30	0.52
Q60	0.32	0.47
Factor name	Time management and study environment	Interpersonal interaction and help-seeking

In brief, this study established a mathematics SDL scale encompassing four subscales and 50 items. EFA revealed that the scale consisted of 12 factors. Table 6 demonstrates the dimensions, factors, and number of the items in the scale.

**Table 6:** Overview of the mathematics SDL scale: Dimensions, factors, contents, and number of items.

Dimension	Factors	Contents	Items no.
Learning motivation	Self-efficacy	Individual’s belief in their own ability to successfully perform a task or achieve a goal.	18
	Identified motivation	Individuals consciously acknowledge the value and relevance of a particular activity to their personal goals or well-being, even if the activity itself is not inherently enjoyable.	
	Intrinsic motivation	Individuals engage in an activity or behavior because it is inherently satisfying, enjoyable, or interesting to the individuals.	
	Extrinsic motivation(Work values)	Individuals perform a task or job because of external rewards or outcomes, such as pay, recognition, or promotions.	
	Motivation (Achievement-oriented)	Individuals are driven by an internal desire to outperform or succeed rather than external rewards.	
Learning strategy	Refined strategies	The process of improving or adapting existing methods, approaches, or plans to better achieve a desired goal or outcome.	8
	Rehearsal strategies	The techniques or methods that individuals use to improve their memory and recall of information.	
	Critical thinking strategies	A set of cognitive skills applied to evaluate and analyze information, arguments, and evidence in order to make informed decisions or form well-reasoned opinions.	
Self-regulation strategy	Self-regulation in problem-solving	Individuals’ ability to manage their thoughts and behaviors in order to effectively solve a problem or overcome a challenge.	17
	SRL in mathematics	Individuals’ ability to manage their own learning process in mathematics, including setting goals, monitoring progress, and adapting strategies to improve understanding and learning performance.	
Learning resource management	Time management and study environment	The process of organizing, planning, and allocating one’s time and study environment effectively	7
	Interpersonal interaction and help-seeking	Engaging with instructors and classmates to collaboratively learn and soliciting assistance or guidance to improve understanding of mathematical concepts.	

### 4.4. Confirmatory factor analysis

Before initiating the confirmatory factor analysis, the data was scrutinized to determine whether the data exhibit multivariate normality. Bollen (1989) contends that for data to be deemed multivariate normal, the multivariate kurtosis (i.e., Mardia’s coefficient), must not exceed p×(p+2) , where *p* represents the number of observed items, and the critical ratio should be confined to a range of 5 or less (Kline, 2011). In this study, Mardia’s coefficient is 734.113, considerably below the threshold of 2,600 ( 50×(50+2) ); however, the critical ratio surpasses 5 with a value of 114.613. Considering the inherent robustness of maximum likelihood estimation in the face of minor deviations from multivariate normality, the technique remains reliable and valid, even when the data contradict statistical suppositions. Consequently, utilizing maximum likelihood estimation within this study retains its trustworthiness.

#### 4.4.1. Competition model

Because each of the 12 latent variables corresponds to an item, the model can be considered a first-order measurement model with 12 latent variables. However, the correlation between the latent variables should be analyzed, and whether the 12 latent variables can be explained by a higher-order variable should be confirmed. To address the above problem, Noar (2003) suggested adopting a competition model, indicated that the best model can be selected through the comparison of the competition models (null, single factor, multifactor orthogonal, multifactor oblique, and second-order factor models). Because EFA was used to identify the 12 latent variables, this study only compared the multifactor orthogonal, multifactor oblique, and second-order factor models to select the best model for validating the mathematics SDL scale.

This study compared the models using the calibration sample (*N* = 507). The results are shown in Table 7. The multifactor orthogonal model had the worst fit. Indices such as *χ*^2^, *χ*^2^/*df*, root mean square error of approximation (RMSEA), comparative fit index (CFI), NNFI (non-normed fit index), SRMR (standardized root mean square), and GFI (goodness of fit index) did not satisfy the criteria, while ECVI (Expected Cross-Validation Index), AIC (Akaike Information Criterion), and BIC (Bayesian Information Criterion) were relatively large (17.98, 9098.67, and 9521.52). In terms of the multifactor oblique model, *χ*^2^, χ^2^/df, RMSEA, CFI, NNFI, SRMR, and GFI did not satisfy the criteria, and ECVI, AIC, and BIC were still relatively large despite the reduction (14.89, 7534.03, and 7985.25).

In terms of the second-order factor model, the results showed that although *χ*^2^ was significant, *χ*^2^/df was only 3.46, which reached the loose standard (the general standard requires less than three and the loose standard requires less than five). Furthermore, the RMSEA, CFI, NNFI, and SRMR reached an acceptable level. The GFI was not ideal but understandable because there were 50 observed variables. Moreover, the ECVI, AIC, and BIC were the smallest (8.40, 4252.17, 4725.76) among the three models, indicating that the second-order factor model had the best fit. In summary, the second-order factor model is a more appropriate validation model for the mathematics SDL scale.

#### 4.4.2. Second-order factor model

The model fit indices of the second-order CFA model with 12 latent variables are listed in Table 8. As mentioned in the previous section, the important fit indices such as RMSEA = 0.070 and *χ*^2^/df = 3.46 were good or acceptable, and the incremental fit indices such as CFI, NFI, and NNFI were acceptable, and the parsimonious fit indices such as PGFI and PNFI were good. Huang (2007) suggested the use of the majority rule to evaluate a model. Because this model has many variables and achieving good results in all indices is difficult, the fitness of the proposed second-order CFA model of mathematics SDL scale is acceptable.

**Table 7:** Evaluation of the fitness of the competition models (calibration sample *N* = 507).

Index model	*χ*^2^(*df*)	*χ*^2^/*df*	RMSEA	CFI	NNFI	SRMR	GFI	ECVI	AIC	BIC
Multifactor orthogonal model	8898.67 (1175)	7.57	0.114	0.63	0.61	0.354	0.45	17.98	9098.67	9521.52
Multifactor oblique model	7322.03 (1169)	6.26	0.102	0.70	0.69	0.102	0.45	14.89	7534.03	7985.25
Second-order factor model	4028.17 (1163)	3.46	0.070	0.86	0.85	0.087	0.69	8.40	4252.17	4725.76

Table 9 shows that the reliability (*R*^2^) of individual observed variables was between 0.33 and 0.85, which satisfied the requirement that the reliability of individual observed variables should be greater than 0.20 (Jöreskog and Sörbom, 1989). This result indicated that the observed variables had good reliability. The composite reliability of the latent variables was between 0.73 and 0.96, which satisfied the requirement that the reliability should be above 0.60 (Fornell and Larcker, 1981). This indicated that the composite reliability is acceptable.

**Table 8:** Overall fitness of the modified model of the mathematics SDL scale (measured sample *N* = 507).

Overall fit index	Evaluation standard	Model index	Evaluation result
Absolute fit index			
Likelihood-ratio *χ*^2^	*p* ≧ 0.05	4028.17^***^	
GFI	≧ 0.90 or 0.80	0.69	Poor
AGFI	≧ 0.90 or 0.80	0.66	Poor
SRMR	≦ 0.08	0.087	Fair
RMSEA	≦ 0.08	0.070	Good
Incremental fit index			
NFI	≧ 0.90	0.82	Fair
NNFI	≧ 0.90	0.85	Fair
RFI	≧ 0.90	0.81	Fair
IFI	≧ 0.90	0.86	Fair
CFI	≧ 0.90	0.86	Fair
Parsimonious fit index			
PGFI	≧ 0.50	0.63	Good
PNFI	≧ 0.50	0.77	Good
PCFI	≧ 0.50	0.82	Good
Likelihood-ratio *χ*^2^/*df*	≦ 3 or ≦ 5(loose standard)	3.46	Acceptable

The evaluation standard of model fit indices is based on Huang (2007) and Doll et al. (1994).

^***^p < 0.001.

Furthermore, Table 9 shows that the standardized factor loadings of all observed variables and the corresponding latent variables were between 0.57 and 0.92. The standardized loadings of all observed variables were above the threshold of 0.45 (Jöreskog and Sörbom, 1989). This indicated that the observed variables were sufficient to reflect the constructed latent variables. The average variance extracted (AVE) of the latent variables is between 0.47 and 0.78, indicating that the contribution of the observed variables to the latent variables was not inferior to the contribution of the error (Fornell and Larcker, 1981).

### 4.5. Cross-validation

In this stage, this study examined the stability of the model, that is, to measure the stability of the calibration sample and validation sample through cross-validation. First, this study used a tight strategy, directly applied the model of the calibration sample (*N*_1_ = 507) to the validation sample (*N*_2_ = 507) and examined whether the factor loadings and covariance between latent variables were the same. Second, this study examined whether the MMF chi-square values (minimum fit function *χ*^2^) of the loose strategy (factor loading and covariance between latent variables were freely estimated) and the tight strategy were significant.

The results (as shown in Table 10) indicated that for the tight strategy, the *χ*^2^ of the validation sample (*N*_2_ = 507) was 4603.49, and its ratio was 53.47%. This indicated that the contribution of the validation sample (*N*_2_ = 507) was slightly higher than that of the calibration sample (*N*_1_ = 507). The test results showed that the model can be applied to different samples in the same population, which means that the proposed model is validated.

**Table 9:** Factor loading, reliability, and average variance explained of the mathematics SDL scale (calibration sample *N* = 507).

Latent variable	Observed variable	Standardized factor loading	*t*-value	Reliability of individual item(*R*^2^)	CR	AVE (%)
SDL in mathematics (Second-order latent variables)	Intrinsic motivation	0.78	18.75^***^	0.61	0.96	0.67
	Extrinsic motivation 1	0.76	16.81^***^	0.58		
	Extrinsic motivation 2	0.80	16.27^***^	0.64		
	Identified motivation	0.90	17.35^^***^^	0.82		
	Self-efficacy	0.55	12.29^***^	0.30		
	Time management and study environment	0.77	15.65^***^	0.59		
	Interpersonal interaction and help-seeking	0.93	16.54^***^	0.87		
	SRL in mathematics	0.90	15.10^***^	0.81		
	Self-regulation in problem-solving	0.87	20.69^***^	0.76		
	Rehearsal strategies	0.55	11.40^***^	0.30		
	Refined strategies	0.97	15.81^***^	0.94		
	Critical thinking strategies	0.89	20.80^***^	0.79		
Intrinsic motivation	A1	0.92	–	0.85	0.86	0.61
	A2	0.71	19.32^***^	0.50		
	A4	0.87	27.48^***^	0.76		
	A6	0.59	14.90^***^	0.35		
Extrinsic motivation (Work values)	A7	0.85	–	0.73	0.90	0.69
	A8	0.88	24.97^***^	0.77		
	A9	0.87	24.48^***^	0.75		
	A10	0.71	18.10^***^	0.50		
Extrinsic motivation (Achievement-oriented)	A11	0.80	–	0.64	0.83	0.63
	A12	0.82	18.86^***^	0.67		
	A13	0.76	17.35^***^	0.57		
Identified motivation	A14	0.75	–	0.57	0.81	0.58
	A17	0.75	16.61^***^	0.57		
	A18	0.79	17.42^***^	0.62		
Self-efficacy	A21	0.91	–	0.82	0.93	0.78
	A22	0.90	31.53^***^	0.82		
	A23	0.88	29.65^***^	0.77		
	A24	0.85	27.20^***^	0.72		
Rehearsal strategies	A25	0.87	–	0.75	0.83	0.62
	A26	0.82	18.13^***^	0.67		
	A27	0.65	14.67^***^	0.42		
Refined strategies	A28	0.66	–	0.43	0.76	0.51
	A29	0.74	14.67^***^	0.55		
	A31	0.74	14.53^***^	0.54		
Critical thinking strategies	A32	0.88	–	0.77	0.85	0.74
	A33	0.85	23.45^***^	0.72		
SRL in mathematics	A35	0.66	–	0.43	0.90	0.57
	A 37	0.69	13.78^***^	0.47		
	A 38	0.61	12.45^***^	0.37		
	A 40	0.83	16.21^***^	0.69		
	A 41	0.80	15.63^***^	0.63		
	A 42	0.84	16.35^***^	0.71		
	A 43	0.82	16.01^***^	0.67		
Self-regulation in problem-solving	A 45	0.87	–	0.76	0.96	0.70
	A 46	0.76	21.63^***^	0.58		
	A 47	0.86	26.94^***^	0.74		
	A 48	0.87	27.73^***^	0.76		
	A 49	0.86	26.64^***^	0.73		
	A 50	0.88	28.39^***^	0.78		
	A 51	0.79	23.10^***^	0.63		
	A 52	0.77	21.89^***^	0.59		
	A 53	0.84	25.82^***^	0.71		
	A 54	0.85	26.17^***^	0.72		
Time management and study environment	A55	0.80	–	0.64	0.82	0.53
	A56	0.88	20.51^***^	0.77		
	A57	0.63	14.19^***^	0.39		
	A59	0.57	12.89^***^	0.33		
Interpersonal interaction and help-seeking	A60	0.71	–	0.50	0.73	0.47
	A62	0.63	13.05^***^	0.39		
	A63	0.72	14.91^***^	0.52		

CR is composite reliability; AVE is average variance extracted.

^***^p < 0.001.

In addition, because of the nested relation between loose and tight strategies, a chi-square difference test was performed. Table 10 shows that under the loose strategy, the MFF*χ*^2^ of the validation sample was 4545.63, which was 57.86 less than the MFF*χ*^2^ of the validation sample under the tight strategy. However, it was not significant when the degree of freedom was 50, indicating that the cross-validation was supported.

### 4.6. Measurement invariance tests

This study used the chi-square difference test, ΔRMSEA, ΔSRMR, and ΔCFI to test the measurement invariance of the measurement model across gender and grade. If Δ*χ*2 is not significant, it means that the measurement model is invariant. However, the chi-square difference is likely to become significant as the sample size increases. Therefore, according to scholars’ recommendations, this study selected the model fit indices such as ΔRMSEA and ΔCFI as the evaluation criteria. When ΔRMSEA <0.01, ΔCFI < 0.01, and ΔSRMR <0.03, it means that factor loading is invariant (Chen, 2007; Wu et al., 2015). When ΔRMSEA <0.01, ΔCFI < 0.01, and ΔSRMR <0.01, it means intercept and residual are invariant (Chen, 2007; Wu et al., 2015). Table 11 shows that the fitness of the baseline model was acceptable (gender: *χ*^2^ = 9031.18, RMSEA = 0.053, SRMR = 0.120, and CFI = 0.839; grade: *χ*^2^ = 9018.61, RMSEA = 0.053, SRMR = 0.091, and CFI = 0.839).

**Table 10:** Summary of cross validation of the model for the mathematics SDL scale (validation sample *N*_2_ = 507).

Compared strategy	Model fitness MMF*χ*^2^ (*df*)	Validation sample MMF*χ*^2^	ΔMFF*χ*^2^	*χ*^2^ Ratio
Loose strategy	8573.79 (2326)	4545.63	57.86 (50)	53.02%
Tight strategy	8609.51 (2376)	4603.49		53.47%

The chi-square difference was less than 67.50 (df = 50); thus, it was not significant at the 5% level.

Second, Model 2 was constructed to determine whether the factor loadings of the two samples were constant. Table 12 shows that the chi-square difference (Δ*χ*^2^ = 87.67) between Gender Model 2 and Model 1 was significant. However, ΔRMSEA = 0.000 was less than 0.01, ΔSRMR = −0.001 was less than 0.03, and ΔCFI = −0.002 was less than 0.01, indicating that the proposed scale had a constant factor loading across gender groups. In addition, the chi-square difference between Grade Model 2 and Model 1 (Δ*χ*^2^ = 22.78) was not significant. ΔRMSEA = 0.000 was less than 0.01, ΔSRMR = 0.001 was less than 0.03, and ΔCFI = 0.000 was less than 0.01, indicating that the proposed scale had a constant factor loading across grade groups.

**Table 11:** Summary of model fit indices of mathematics SDL scale (*N* = 1,014).

	*χ* ^2^	*df*	*p*-value	RMSEA	SRMR	CFI
*Gender*						
M1: Baseline model	9031.18	2,326	0.000	0.053	0.120	0.839
M2: Constant factor loading	9118.84	2,364	0.000	0.053	0.119	0.837
M3: Constant structural weight	9192.04	2,376	0.000	0.053	0.145	0.836
M4: Residual invariant	9451.04	2,438	0.000	0.053	0.144	0.831
*Grade*						
M1: Baseline model	9018.61	2,326	0.000	0.053	0.091	0.839
M2:Constant factor loading	9041.39	2,364	0.000	0.053	0.092	0.839
M3:Constant structural weight	9051.74	2,376	0.000	0.053	0.093	0.840
M4: Residual invariant	9189.72	2,438	0.000	0.052	0.093	0.838

Third, based on the constant factor loading, Model 3 was constructed to test whether the structural weights of the two samples were constant and whether the structural weight was constant across gender and grade groups. The chi-square difference (Δ*χ*2 = 73.20) between Gender Models 3 and 2 was significant, but ΔRMSEA = 0.000 was less than 0.01, ΔSRMR = 0.026 was less than 0.03, and ΔCFI = −0.001 was less than 0.01, indicating that this scale had constant structural weight across gender groups. In addition, the chi-square difference (Δ*χ*^2^ = 10.36) between Grade Models 3 and 2 was not significant, ΔRMSEA = 0.000 was less than 0.01, ΔSRMR = 0.001 was less than 0.01, and ΔCFI = 0.001 was less than 0.01, indicating that this scale had constant structural weight across grade groups. Fourth, based on the constant factor loading, Model 4 was constructed to test whether the residuals of the two samples were constant and whether the residuals were constant across gender and grade groups. The chi-square difference (Δ*χ*2 = 82.41) between Gender Models 4 and 3 was significant, but ΔRMSEA = 0.000 was less than 0.01, ΔSRMR = −0.001 was less than 0.01, and ΔCFI = −0.005 was less than 0.01, indicating that this scale had residual invariance across gender groups. In addition, the chi-square difference (Δ*χ*^2^ = 137.98) between Grade Models 4 and 3 was significant, but ΔRMSEA = −0.001 was less than 0.01, ΔSRMR = 0.000 was less than 0.01, and ΔCFI = - 0.002 was less than 0.01, indicating that this scale had residual invariance across grade groups. In conclusion, in terms of the measurement model, this scale has strong measurement invariance across gender and grade groups.

### 4.7. Analysis of latent mean differences between different gender and grade groups

This study compared the difference between male and female students and the difference among students in grades seven, eight, and nine in their answers to the mathematics SDL scale. As shown in Table 13, the difference in total score between gender groups was significant in the *t*-test (*p* < 0.001), indicating that in terms of mathematics SDL abilities, male students (*M* = 135.97) have higher perception scores than female students (*M* = 132.47).

**Table 12:** Summary of model invariance across gender and grade (*N* = 1,014).

	Δ*χ*^2^	Δ*df*	*p*-value	ΔRMSEA	ΔSRMR	ΔCFI
*Gender model comparison*						
M2 vs. M1	87.67	38	0.000	0.000	−0.001	−0.002
M3 vs. M2	73.20	12	0.000	0.000	0.026	−0.001
M4 vs. M3	82.41	12	0.000	0.000	−0.001	−0.005
*Grade model comparison*						
M2 vs. M1	22.78	38	0.976	0.000	0.001	0.000
M3 vs. M2	10.36	12	0.585	0.000	0.001	0.001
M4 vs. M3	137.98	62	0.000	−0.001	0.000	−0.002

After examining the performance of male and female students on various factors of SDL in mathematics, we observed that male students had a significantly higher latent means than female students in terms of many factors of SDL in mathematics, including intrinsic motivation, extrinsic motivation (work values), self-efficacy, refined strategies, critical thinking strategies, and time management and study environment. The difference in self-efficacy was the largest (ΔM = 0.41), followed by intrinsic motivation (ΔM = 0.24). The latent mean of identified motivation of female students was slightly higher than that of male students.

In addition, the F-test showed that the mathematics SDL scores of students in different grades were significantly different, as shown in Table 14. The total score of the seventh grade (*M* = 137.69) was significantly higher than that of the eighth grade (*M* = 133.33) and ninth grade (*M* = 131.77). Although the score of the eighth grade was higher than that of the ninth grade, it was still not significant.

**Table 13:** Summary of differences in various factors across genders.

Factor	Gender	Number of students	Mean	Standard deviation	*t*-value	*p*-value
Intrinsic motivation	Male	3,177	2.64	0.79	12.08^***^	<0.001
	Female	2,960	2.40	0.71		
Extrinsic motivation (Work values)	Male	3,177	2.87	0.76	5.67^***^	<0.001
	Female	2,960	2.76	0.71		
Extrinsic motivation (Achievement-oriented)	Male	3,177	3.06	0.75	−0.78	0.435
	Female	2,960	3.07	0.70		
Identified motivation	Male	3,177	2.83	0.77	−2.83^**^	0.005
	Female	2,960	2.88	0.69		
Self-efficacy	Male	3,177	2.42	0.88	19.30^***^	<0.001
	Female	2,960	2.01	0.78		
Rehearsal strategies	Male	3,177	2.29	0.77	−1.41	0.159
	Female	2,960	2.32	0.69		
Refined strategies	Male	3,177	2.61	0.79	4.23^***^	<0.001
	Female	2,960	2.53	0.70		
Critical thinking strategies	Male	3,177	2.90	0.80	4.57^***^	<0.001
	Female	2,960	2.81	0.73		
Self-regulation in problem-solving	Male	3,177	2.95	0.71	−0.01	0.992
	Female	2,960	2.95	0.62		
SRL in mathematics	Male	3,177	2.65	0.74	1.90	0.058
	Female	2,960	2.61	0.64		
Time management and study environment	Male	3,177	2.49	0.77	2.67^**^	0.008
	Female	2,960	2.44	0.65		
Interpersonal interaction and help-seeking	Male	3,177	2.72	0.77	−1.07	0.283
	Female	2,960	2.74	0.68		
Total score	Male	3,177	135.97	31.43	4.70^***^	<0.001
	Female	2,960	132.47	26.74		

^*^p < 0.05; ^**^p < 0.01; ^***^p < 0.001.

After examining the latent mean difference of various factors of SDL in mathematics among students of different grades, we observed that most of the factors of the seventh graders were significantly higher than those of the eighth and ninth graders, such as extrinsic motivation-work values (2.96, 2.78, 2.71), extrinsic motivation (achievement-oriented) (3.15, 3.02, 3.03), identified motivation (2.94, 2.83, 2.78), rehearsal strategies (2.42, 2.27, 2.22), critical thinking strategies (2.94, 2.82, 2.82), self-regulation in problem-solving (3.02, 2.92, 2.91), SRL in mathematics (2.72, 2.62, 2.55), and time management and study environment (2.54, 2.46, 2.39). Some factors of the eighth grader are higher than those of the ninth grade, such as extrinsic motivation (work values), SRL in mathematics, and time management and study environment. In summary, mathematics SDL ability does not improve with increasing grade level (see Table 14).

**Table 14:** Summary of differences in various factors across grades.

Factor	Grade	Number of students	Mean	Standard deviation	*F*-score	*p*-value	Scheffe post-hoc comparison
Intrinsic motivation	(1) Seventh grade	2,041	2.55	0.75	1.43	0.238	
	(2) Eighth grade	2,146	2.51	0.77			
	(3) Ninth grade	1,950	2.52	0.76			
Extrinsic motivation (Work values)	(1) Seventh grade	2,041	2.96	0.72	64.54^***^	<0.001	1 > 2 > 3
	(2) Eighth grade	2,146	2.78	0.74			
	(3) Ninth grade	1,950	2.71	0.74			
Extrinsic motivation (Achievement-oriented)	(1) Seventh grade	2,041	3.15	0.68	21.22^***^	<0.001	1 > 2,3
	(2) Eighth grade	2,146	3.02	0.73			
	(3) Ninth grade	1,950	3.03	0.76			
Identified motivation	(1) Seventh grade	2,041	2.94	0.71	23.02^***^	<0.001	1 > 2,3
	(2) Eighth grade	2,146	2.83	0.73			
	(3) Ninth grade	1,950	2.78	0.74			
Self-efficacy	(1) Seventh grade	2,041	2.21	0.85	0.40	0.671	
	(2) Eighth grade	2,146	2.23	0.85			
	(3) Ninth grade	1,950	2.22	0.87			
Rehearsal strategies	(1) Seventh grade	2,041	2.42	0.74	41.26^***^	<0.001	1 > 2,3
	(2) Eighth grade	2,146	2.27	0.73			
	(3) Ninth grade	1,950	2.22	0.72			
Refined strategies	(1) Seventh grade	2,041	2.62	0.74	7.23^**^	0.001	1 > 3
	(2) Eighth grade	2,146	2.55	0.74			
	(3) Ninth grade	1,950	2.53	0.76			
Critical thinking strategies	(1) Seventh grade	2,041	2.94	0.75	15.35^***^	<0.001	1 > 2,3
	(2) Eighth grade	2,146	2.82	0.76			
	(3) Ninth grade	1,950	2.82	0.78			
Self-regulation in problem-solving	(1) Seventh grade	2,041	3.02	0.65	15.51^***^	<0.001	1 > 2,3
	(2) Eighth grade	2,146	2.92	0.67			
	(3) Ninth grade	1,950	2.91	0.68			
SRL in mathematics	(1) Seventh grade	2,041	2.72	0.69	28.10^***^	<0.001	1 > 2 > 3
	(2) Eighth grade	2,146	2.62	0.70			
	(3) Ninth grade	1,950	2.55	0.69			
Time management and study environment	(1) Seventh grade	2,041	2.54	0.73	21.32^***^	<0.001	1 > 2 > 3
	(2) Eighth grade	2,146	2.46	0.70			
	(3) Ninth grade	1,950	2.39	0.71			
Interpersonal interaction and help-seeking	(1) Seventh grade	2,041	2.75	0.74	1.71	0.180	
	(2) Eighth grade	2,146	2.73	0.72			
	(3) Ninth grade	1,950	2.70	0.73			
Total score	(1) Seventh grade	2,041	137.69	28.41	22.24^***^	<0.001	1 > 2,3
	(2) Eighth grade	2,146	133.33	29.47			
	(3) Ninth grade	1,950	131.77	29.75			

^*^p < 0.05; ^**^p < 0.01; ^***^p < 0.001.

## 5. Conclusions and recommendations

This study established a mathematics SDL scale to investigate the SDL performance of Taiwan junior high school students 3 years after the implementation of the new curriculum guideline and to understand the differences across genders and grades. First, in terms of reliability analysis, the composite reliabilities of the 12 factors are between 0.73 and 0.96 and are all above 0.60, indicating that the scale has good composite reliability. In terms of validity analysis, after expert review, the items of the scale passed the I-CVI and S-CVI standards; therefore, the scale has content validity. This study conducted an EFA using a pretest sample of 681 students to establish the final items of the mathematics SDL scale. Subsequently, this study used a final sample of 5,456 junior high school students for CFA, cross-validation, and measurement invariance tests across genders and grades. The CFA indicated that the second-order model fit the observed data well. In other words, the 12 factors under the four dimensions, namely learning motivation, learning strategies, mathematical self-regulation, and learning resource management can be explained by the SDL in mathematics.

Second, the cross-validation indicated that the scale’s model has strong stability. Third, the measurement invariance tests across genders and grades indicated that the measurement model of this scale had strong measurement invariance across gender and grade groups. The results of this study demonstrated the richness and complexity of SDL in mathematics. Follow-up studies can be conducted in other countries or regions that promote SDL curriculum reform to confirm the reliability and validity of this scale.

The researcher examined the latent mean differences in mathematics SDL abilities by gender and grade based on the answers of 6,137 students in the pretest and formal test. The analysis results indicated that there is a gender difference in mathematics SDL. Male students are higher than female students in many factors, with the greatest difference being in intrinsic motivation and self-efficacy. This finding is similar to PISA’s (OECD, 2014) study on Taiwanese students’ motivation and beliefs in mathematics learning. Because the PISA survey was conducted before the implementation of the new curriculum guideline, the findings implied that gender differences in mathematics learning are not effectively addressed by the new curriculum guideline. It is worth noting that male and female students have no differences in innate mathematical abilities (Lindberg et al., 2010), they have similar mathematical attitudes (Ghasemi and Burley, 2019), and the difference is primarily owing to sociocultural factors (Kane and Mertz, 2012). A myth remains in Taiwan’s culture that men are suitable for studying science and technology and women are suitable for studying humanities, and women are not encouraged to study STEM-related subjects and work in related industries. Although Taiwan ranks first in Asia in terms of the gender equity index (Executive Yuan, 2022) and the mathematics curriculum encourages the inclusion of gender equality, the findings of PISA and this study suggest that there is still scope for improvement in mathematics education in Taiwan in terms of gender equity. In addition, the SDL in mathematics does not increase with grade. This result is similar to that of Mok et al. (2007) and different from that of Chen et al. (2021). This shows that although the new wave of curriculum reform in Taiwan has contributed to improving the SDL of the next generation, it is not reflected in the subject learning.

These findings implied the importance of teacher education for developing students’ SDL. Although much research has been conducted on SDL from the student perspective, more research on how teachers can guide students in SDL is needed (Dignath and Büttner, 2018). Subsequent studies could explore how mathematics teachers facilitate girls or higher graders to develop self-efficacy in mathematics and intrigue their intrinsic motivation. Take the item, I have always believed that mathematics is one of my best subjects, as an example. This assertion underscores the necessity to foster a positive self-perception in girls and elder students, empowering them to excel in mathematical disciplines. In addition, future studies could consider the dual role (Kramarski, 2018) that teachers play in SDL professional development and SDL enhancement. These studies may contribute to a more diverse and enriched field of study.

## Data availability statement

The original contributions presented in the study are included in the article/supplementary material, further inquiries can be directed to the corresponding author.

## Ethics statement

The studies involving human participants were reviewed and approved by the Research Ethics Committee of National Taiwan Normal University (NTNUREC-202005ES024). Written informed consent to participate in this study was provided by the participants' legal guardian/next of kin.

## Author contributions

C-HL and C-HC contributed to the conceptualization and design of the study. C-HC collected the data and performed the statistical analysis. C-HL wrote the first draft of the manuscript. C-HC reviewed and edited the manuscript. All authors contributed to the article and approved the submitted version.

## Funding

This work was supported by Taiwan’s National Science and Technology Council under Grant numbers (MOST 109-2511-H-656 -001 -MY3 and MOST 111-2410-H-142-003). The funding agencies had no involvement in this research.

## Conflict of interest

The authors declare that the research was conducted in the absence of any commercial or financial relationships that could be construed as a potential conflict of interest.

## Publisher’s note

All claims expressed in this article are solely those of the authors and do not necessarily represent those of their affiliated organizations, or those of the publisher, the editors and the reviewers. Any product that may be evaluated in this article, or claim that may be made by its manufacturer, is not guaranteed or endorsed by the publisher.

## References

[ref1] Amey B. E. (2008). An exploration of the relationship between experiential learning and self-directed learning readiness. (Doctoral dissertation). Boca Raton, FL: Florida Atlantic University.

[ref3] Bandura's A. (1977). Self-efficacy: toward a unifying theory of behavioral change. Psychol. Rev. 84, 191–215. doi: 10.1037/0033-295X.84.2.191, PMID: 847061

[ref4] Bentler P. M. Bonett D. G. (1980). Significance tests and goodness-of-fit in the analysis of covariance structures. Psychol. Bull. 88, 588–606. doi: 10.1037/0033-2909.88.3.588

[ref5] Bentler P. M. Chou C. P. (1987). Practical issues in structural modeling. Sociol. Methodol. Res. 16, 78–117. doi: 10.1177/0049124187016001004

[ref6] Bollen K. A. (1989). Structural equations with latent variables. New York, NY: John Wiley & Sons.

[ref7] Brandt W. C. (2020). Measuring student success skills: a review of the literature on self-directed learning. 21st century success skills. Dover, NH: National Center for the Improvement of Educational Assessment.

[ref8] Chen F. F. (2007). Sensitivity of goodness of fit indexes to lack of measurement invariance. Struct. Equ. Modeling 14, 464–504. doi: 10.1080/10705510701301834

[ref9] Chen C. C. Chang C. H. (2007). Quantitative research and statistical analysis. Taipei, Taiwan: New Sharing.

[ref10] Chen C.-H. Chen K.-Z. Tsai H.-F. (2021). Did self-directed learning curriculum guidelines change Taiwanese high-school students’ self-directed learning readiness? Asia-Pac. Educ. Res. 31, 409–426. doi: 10.1007/s40299-021-00582-w

[ref11] Cheng S.-F. Kuo C.-L. Lin K.-C. Hsieh J.-L. (2010). Development and preliminary testing of a self-rating instrument to measure self-directed learning ability of nursing students. Int. J. Nurs. Stud. 47, 1152–1158. doi: 10.1016/j.ijnurstu.2010.02.002, PMID: 20223455

[ref12] Corno L. (2001). “Volitional aspects of self-regulated learning” in Self-regulated learning and academic achievement: Theoretical perspectives. eds. Zimmerman B. J. Schunk D. H.. 2nd ed (Hillsdale, NJ: Erlbaum), 191–225.

[ref13] Crocker L. Algina J. (2006). Introduction to classical and modern test theory. Mason, Ohio: Cengage Learning.

[ref14] Dalkey N. C. (1969). The Delphi method: An experimental study of group opinion. Santa Monica, CA: Rand Corp.

[ref15] de Bruin A. B. H. van Merriënboer J. J. G. (2017). Bridging cognitive load and self-regulated learning research: a complementary approach to contemporary issues in educational research. Learn. Instr. 51, 1–9. doi: 10.1016/j.learninstruc.2017.06.001

[ref16] Delbecq A. L. Van de Ven A. H. Gustafson D. H. (1975). Group techniques for program planning: A guide to nominal group and Delphi processes. Glenview, IL: Scott, Foresman.

[ref17] DeVellis R. F. (1998). Scale development: Theory and applications. CA: Sage.

[ref18] Dignath C. Buettner G. Langfeldt H. (2008). How can primary school students learn self-regulated learning strategies most effectively? A meta-analysis on self-regulation training programmes. Educ. Res. Rev. 3, 101–129. doi: 10.1016/j.edurev.2008.02.003

[ref002] Dignath C. Büttner G. (2018). Teachers’ direct and indirect promotion of self-regulated learning in primary and Secondary School Mathematics Classes – insights from video-based classroom observations and teacher interviews. Metacognition Learn. 13, 127–157. doi: 10.1007/s11409-018-9181-x

[ref19] Doll W. J. Xia W. Torkzadeh G. (1994). A confirmatory factor analysis of the end-user computing satisfaction instrument. MIS Q. 12, 259–274. doi: 10.2307/249524

[ref20] Fornell C. Larcker D. (1981). Evaluating structural equation models with unobservable variables and measurement error. J. Mark. Res. 18, 39–50. doi: 10.2307/3151312

[ref21] Gabrielle D. M. Guglielmino L. M. Guglielmino P. J. (2006). Developing self-directed learning readiness of future leaders in a military college through instructional innovation. Int. J. Self-Directed Learn. 3, 24–35.

[ref22] Ghasemi E. Burley H. (2019). Gender, affect, and math: a cross-national meta-analysis of trends in international mathematics and science study 2015 outcomes. Large Scale Assess. Educ. 7:10. doi: 10.1186/s40536-019-0078-1

[ref23] Guglielmino L. M. (1977). Development of the self-directed learning readiness scale (doctoral dissertation). Athens, Georgia: University of Georgia. Available at: ProQuest Dissertations and Theses Global database UMI No. 7806004.

[ref24] H.K. Curriculum Development Council (2017). Secondary education curriculum guide draft booklet 3 effective learning and teaching: developing lifelong and self-directed learners. Available at: http://www.edb.gov.hk/attachment/en/curriculum-development/renewal/Guides/SECG%20booklet%203_20170531.pdf

[ref25] Huang F. M. (2007). Theory and application of structural equation models (5th). Taipei, Taiwan: Wu-Nan Press.

[ref26] Jöreskog K. G. Sörbom D. (1989). LISREL 7: a guide to the program and applications. Chicago, IL: SPSS.

[ref27] Kane J. M. Mertz J. E. (2012). Debunking myths about gender and mathematics performance. Not. AMS 59, 10–21. doi: 10.1090/noti790

[ref28] Kirschner P. A. Hendrick C. (2020). How learning happens: Seminal works in educational psychology and what they mean in practice. New York, NY: Routledge.

[ref29] Kline R. B. (2011). Principles and practice of structural equation modeling (3rd). New York, NY: Guilford Press.

[ref30] Knowles M. (1975). Self-directed learning: A guide for learners and teachers. New York, NY: Cambridge Books.

[ref31] Kramarski B. (2018). “Teachers as agents in promoting students' SRL and performance” in Handbook of self-regulation of learning and performance. eds. Schunk D. H. Greene J. A. (New York, NY: Routledge), 223–239.

[ref32] Lindberg S. M. Hyde J. S. Petersen J. L. Linn M. C. (2010). New trends in gender and mathematics performance: a meta-analysis. Psychol. Bull. 136, 1123–1135. doi: 10.1037/a0021276, PMID: 21038941 PMC3057475

[ref33] Marsh H. W. Balla J. R. McDonald R. P. (1988). Goodness-of-fit indexes in confirmatory factor analysis: the effect of sample size. Psychol. Bull. 103, 391–410. doi: 10.1037/0033-2909.103.3.391

[ref34] Marsh H. W. Hocevar D. (1985). Application of confirmatory factor analysis to the study of self-concept: first- and higher order factor models and their invariance across groups. Psychol. Bull. 97, 562–582. doi: 10.1037/0033-2909.97.3.562

[ref35] Mevarech Z. R. Kramarski B. (2014). Critical maths for innovative societies: The role of metacognitive pedagogies on mathematical reasoning. Paris, France: OECD.

[ref36] Mevarech Z. R. Verschaffel L. De Corte E. (2018). “Metacognitive pedagogies in mathematics classrooms: from kindergarten to college and beyond” in Handbook of self-regulation of learning and performance. eds. Schunk D. H. Greene J. A. (New York, NY: Routledge/Taylor and Francis Group), 109–123.

[ref37] Ministry of Education (2018). Mathematics curriculum guidelines of 12-year basic education for elementary and secondary schools and general senior high schools. Taipei, Taiwan.

[ref38] Mok M. M. C. Cheng Y. C. Leung S. O. Shan P. W. J. Moore P. Kennedy K. (2007). “Self-directed learning as a key approach to effectiveness of education: a comparison among mainland China, Hong Kong, Macau, and Taiwan” in International handbook of school effectiveness and improvement. ed. Townsend T. (Dordrecht, Netherlands: Springer), 839–858.

[ref39] National Academies of Sciences, Engineering, and Medicine. (2018). How people learn II: learners, contexts, and cultures. Washington, DC: The National Academies Press.

[ref40] National Academy for Educational Research. (2018). Curriculum guidelines for 12-year basic education: General guidelines. Taipei, Taiwan: Ministry of Education. Available at: https://cirn.moe.edu.tw/Upload/file/32077/83646.pdf

[ref41] New Zealand Ministry of Education. (2007). The New Zealand curriculum. Wellington, NZ: New Zealand.

[ref42] Noar S. M. (2003). The role of structural equation modeling in scale development. Struct. Equ. Modeling 10, 622–647. doi: 10.1207/S15328007SEM1004_8

[ref001] OECD (2014). PISA 2012 Results: What Students Know and Can Do – Student Performance in Mathematics, Reading and Science (Volume I, Revised edition, February 2014), Paris: OECD Publishing. doi: 10.1787/9789264201118-en

[ref43] OECD (2018). PISA 2021Mathematics framework (draft). Available at: https://pisa2021-maths.oecd.org/files/PISA%202021%20Mathematics%20Framework%20Draft.pdf

[ref44] Panadero E. (2017). A review of self-regulated learning: six models and four directions for research. Front. Psychol. 8, 1–28. doi: 10.3389/fpsyg.2017.00422, PMID: 28503157 PMC5408091

[ref45] Pintrich P. R. Smith D. A. F. Garcia T. Mckeachie W. J. (1991). A manual for the use of the motivated strategies for learning questionnaire (MSLQ). Ann Arbor, MI: University of Michigan.

[ref46] Polit D. F. Beck C. T. (2006). The content validity index: are you sure you know what’s being reported? Critique and recommendations. Res. Nurs. Health 29, 489–497. doi: 10.1002/nur.20147, PMID: 16977646

[ref47] Polya G. (1957). How to solve it? (2nd). Princeton: Princeton University Press.

[ref48] Saks K. Leijen Ä. (2014). Distinguishing self-directed and self-regulated learning and measuring them in the e-learning context. Procedia Soc. Behav. Sci. 112, 190–198. doi: 10.1016/j.sbspro.2014.01.1155

[ref49] Schoenfeld's A. H. (1985). Mathematical problem solving. New York: Academic Press.

[ref50] Schoenfeld A. H. (1992). “Learning to think mathematically: problem solving, metacognition, and sense making in mathematics” in Handbook of research on mathematics teaching and learning: A project of the National Council of teachers of mathematics. ed. Grouws D. A. (Reston, VA: National Council of Teachers of Mathematics), 334–370.

[ref51] Schoenfeld A. H. (2014). What makes for powerful classrooms, and how can we support teachers in creating them? A story of research and practice, productively intertwined. Educ. Res. 43, 404–412. doi: 10.3102/0013189X14554450

[ref52] Schunk D. H. (2012). Learning theories: an educational perspective (6th). Boston: Allyn & Bacon.

[ref53] Schunk D. H. (2020). Learning theories: an educational perspective (8th). Boston: Allyn & Bacon.

[ref54] Schunk D. H. Mullen C. A. (2013). “Motivation” in International guide to student achievement. eds. John H. Eric M. A. (New York, NY: Routledge), 67–69.

[ref55] Schunk D. H. Rice J. M. (1987). Enhancing comprehension skill and self-efficacy with strategy value information. J. Read. Behav. 19, 285–302. doi: 10.1080/10862968709547605

[ref56] Schunk D. H. Rice J. M. (1991). Learning goals and progress feedback during reading comprehension instruction. J. Read. Behav. 23, 351–364. doi: 10.1080/10862969109547746

[ref57] Senge P. Cambron-McCabe N. Lucas T. Smith B. Dutton J. Kleiner A. (2000). Schools that learn: a fifth discipline fieldbook for educators, parents, and everyone who cares about education. New York, NY: Doubleday/Currency.

[ref58] Spector P. E. (1992). Summated rating scale construction: An introduction. Thousand Oaks, CA: Sage.

[ref59] Steffens K. (2015). Competences, learning theories and MOOCs: recent developments in lifelong learning. Eur. J. Educ. 50, 41–59. doi: 10.1111/ejed.12102

[ref60] Stillman G. Mevarech Z. R. (2010). Metacognitive research in mathematics education: from hot topic to mature field. ZDM Int. J. Math. Educ. 42, 145–148. doi: 10.1007/s11858-010-0245-x

[ref61] Vermeer H. J. Boekaerts M. Seegers G. (2000). Motivational and gender differences: sixth-grade students’ mathematical problem-solving behavior. J. Educ. Psychol. 92, 308–315. doi: 10.1037/0022-0663.92.2.308

[ref62] Wiliam D. (2018). Embedded formative assessment (2nd). Bloomington, IN: Solution Tree Press.

[ref63] Winne P. H. (1996). A metacognitive view of individual differences in self-regulated learning. Learn. Individ. Differ. 8, 327–353. doi: 10.1016/S1041-6080(96)90022-9

[ref64] Wu M. L. (2012). SPSS operation and application for statistical analysis of questionnaire. Taipei, Taiwan: Wu-Nan Press.

[ref65] Wu T. H. Chang C. C. Chen C. Y. Wang J. D. Lin C.-Y. (2015). Further psychometric evaluation of the self-stigma scale-short: measurement invariance across mental illness and gender. PLoS One 10, 1–12. doi: 10.1371/journal.pone.0117592, PMID: 25659115 PMC4320062

[ref66] Yuan E.. (2022). Gender at a glance in R.O.C. Taipei, Taiwan.

[ref67] Zimmerman's B. J. (2000). “Attaining self-regulation: a social cognitive perspective” in Handbook of self-regulation. eds. Boekaerts M. Pintrich P. R. Zeidner M. (San Diego: Academic Press), 13–39.

[ref68] Zimmerman B. J. (2008). Investigating self-regulation and motivation: historical background, methodological developments, and future prospects. Am. Educ. Res. J. 45, 166–183. doi: 10.3102/0002831207312909

